# Editorial: Addressing the unmet needs of cataract patients: when quality of vision can make the difference in quality of life

**DOI:** 10.3389/fmed.2023.1232243

**Published:** 2023-06-29

**Authors:** Rita Mencucci, Eleonora Favuzza, Filomena Ribeiro

**Affiliations:** ^1^Eye Clinic, Department of Neurosciences, Psychology, Drug Research and Child Health (NEUROFARBA), University of Florence, Florence, Italy; ^2^Department of Ophthalmology, Hospital da Luz, Lisbon, Portugal

**Keywords:** cataract surgery, presbyopia correction, enhanced monofocal intraocular lenses, intraocular lens, multifocal intraocular lens, extended depth of focus (EDOF), quality of vision

“*Senectus ipsa morbus est”* Terentius highlighted already in the second century BC that aging can have a significant impact on the quality of life. This is particularly true for vision, one of the most important among the five senses, together with hearing, for the closest relationship with social life. Even though quality of life and quality of vision are not real synonyms, both can contribute to “wellness” in aging.

A cataract is one of the main causes of visual impairment in old age. Even though clinical interventional studies on these research topics are lacking, it has been suggested that cataract surgery may decrease fall risk, reduce depression, and limit the risk of cognitive impairment (Mencucci et al.). In the narrative review published on this Research Topic (Mencucci et al.), we also emphasize the need to move from the concept of visual acuity to functional vision, especially in the context of the older adult patients. Further studies are necessary in order to evaluate the impact on the cited outcomes of different cataract treatment strategies, such as systematic bilateral vs. monolateral surgery and the use of different intraocular lenses (Mencucci et al.).

In addition to aging, environmental factors, such as UV exposure, diabetes, smoking, and some prescription drugs, can contribute to cataract formation. In particular, the study by Carlson et al. shows how drug-induced cataract represents a poorly addressed source of cataract.

In this context, choosing the right timing of cataract surgery is crucial, and new parameters have been proposed: beyond visual acuity, an objective scatter index can be a helpful early indicator of subjective visual function impairment (Li Y. et al.).

Cataract surgery is a highly successful and cost-effective procedure, even though various factors such as diabetic retinopathy, glaucoma, and at-risk surgery, can affect its outcome (AlRyalat et al.). In the majority of cases, the implantation of conventional monofocal intraocular lenses (IOLs) allows restoration of distance vision, with a very good quality of vision. Nevertheless, these IOLs do not provide spectacle independence in terms of near and intermediate vision, which are involved in many common daily tasks. Therefore, this has led to a growing interest in multifocal IOLs, trifocal IOLs, and extended depth of focus (EDOF) IOLs (1, 2). Although these are good options (Pedrotti et al.), there are possible drawbacks related to photic phenomena, reduced contrast sensitivity (Wang et al.), and reduced stereopsis when a blended or monovision approach is chosen (Zhu et al.).

An important factor that can influence the quality of life after cataract surgery is corneal astigmatism, and among the correction methods (Ding et al.), toric IOLs are the most used and the most successful approach during cataract surgery (3). This strategy has also been applied to multifocal/trifocal/EDOF intraocular lenses (Li Z. et al.).

In recent years, intermediate vision has gained importance, since many daily activities, such as cooking, performing hobbies, and using digital devices may not correlate with far best corrected visual acuity (4). The study by Ribeiro et al. (5) revealed that patients primarily dedicated their time to near (42.53%) and intermediate (30.23%) visual tasks and confirmed the significance of the range of distances between 1 m and ~30–40 cm for the daily life activities.

To reduce the visual disturbances related to trifocal and EDOF IOLs, enhanced monofocal IOLs that give optimal far vision with functional intermediate vision have been introduced. These IOLs demonstrated excellent visual performances, especially at intermediate distances while maintaining good quality of vision, contrast sensitivity, and overall patient satisfaction (6).

Cataract surgery and different IOL options may have a critical influence on visual function, mental and systemic health, and quality of life. Future directions not only in terms of different IOLs but also in determining appropriate instruments to measure the challenge related to different tasks are needed.

## Author contributions

All authors contributed to the conception and design of the editorial, wrote the first draft of the manuscript, manuscript revision, and read and approved the submitted version.

## References

[1] Ferreira TB Ribeiro FJ Silva D Matos AC Gaspar S Almeida S. Comparison of refractive and visual outcomes of 3 presbyopia-correcting intraocular lenses. J Cataract Refract Surg. (2022) 48:280–7. 10.1097/j.jcrs.0000000000000743 34321410

[2] Ribeiro F Ferreira TB. Comparison of clinical outcomes of 3 trifocal IOLs. J Cataract Refract Surg. (2020) 46:1247–52. 10.1097/j.jcrs.0000000000000212 32898095

[3] Mencucci R Giordano C Favuzza E Gicquel JJ Spadea L Menchini U. Astigmatism correction with toric intraocular lenses: wavefrontaberrometry and quality of life. Br J Ophthalmol. (2013) 97:578–82. 10.1136/bjophthalmol-2013-303094 23467788

[4] Ribeiro F Cochener B Kohnen T Mencucci R Katz G Lundstrom M. Definition and clinical relevance of the concept of functional vision in cataract surgery ESCRS Position Statement on Intermediate Vision: ESCRS Functional Vision Working Group. J Cataract Refract Surg. (2020) 46(Suppl.1):S1–3. 10.1097/j.jcrs.0000000000000096 32126025

[5] Ribeiro F Ferreira TB Silva D Matos AC Gaspar S Piñero DP. Analysis of daily visual habits in a presbyopic population. J Ophthalmol. (2023) 2023:6440954. 10.1155/2023/6440954 37089413 PMC10118895

[6] Mencucci R Cennamo M Venturi D Vignapiano R Favuzza E. Visual outcome, optical quality, and patient satisfaction with a new monofocal IOL, enhanced for intermediate vision: preliminary results. J Cataract Refract Surg. (2020) 46:378–87. 10.1097/j.jcrs.0000000000000061 32050218

